# Recurrent pleural effusion: a case of metastatic melanoma 12 years after initial diagnosis

**DOI:** 10.1093/jscr/rjaf144

**Published:** 2025-03-25

**Authors:** Olia Poursina, Susan Karki, Jingxin Qiu

**Affiliations:** Department of Pathology and Laboratory Medicine, Roswell Park Comprehensive Cancer Center, Elm and Carlton St., Buffalo, NY 14263, United States; Department of Pathology and Laboratory Medicine, Roswell Park Comprehensive Cancer Center, Elm and Carlton St., Buffalo, NY 14263, United States; Department of Pathology and Laboratory Medicine, Roswell Park Comprehensive Cancer Center, Elm and Carlton St., Buffalo, NY 14263, United States

**Keywords:** malignant melanoma, metastatic, recurrent pleural effusion, pleural tumors

## Abstract

Pleural effusion caused by metastatic melanoma is uncommon, occurring in only 2% of cases, and is associated with a poor prognosis. Despite its rarity, it is critical to consider melanoma as a potential underlying cause when evaluating pleural effusion, especially in patients with a history of melanoma. Most metastases occur within the first 3–5 years after diagnosis. This case report highlights an unusual presentation of amelanotic pleural effusion that developed 12 years after the initial melanoma diagnosis. The prolonged latency emphasizes the importance of long-term follow-up for patients and maintaining a high suspicion of melanoma when evaluating metastatic pleural effusion.

## Introduction

Melanoma, the fifth most common malignancy in the United States [1], is about only 1% of all skin cancers but is responsible for the highest skin cancer mortality [2]. According to the American Society for Clinical Oncology, metastasis is present in 4% of melanomas [3], and the most common site is the lung. Pleural effusion alone is rare and can be seen in only 2% of cases [4]. It is more common in males over 40 years old [5]. Metastasis to the pleura with a high lactic dehydrogenase (LDH) has the worst prognosis, with a 1-year survival rate of 33% [6]. Most of the metastatic relapses occur within three years, but late metastases can occur even after decades and have a poorer prognosis [7]. Here, we present a case of recurrent pleural effusion from metastatic melanoma after 12 years of diagnosis of the primary tumor.

## Case report

**Figure 1:**
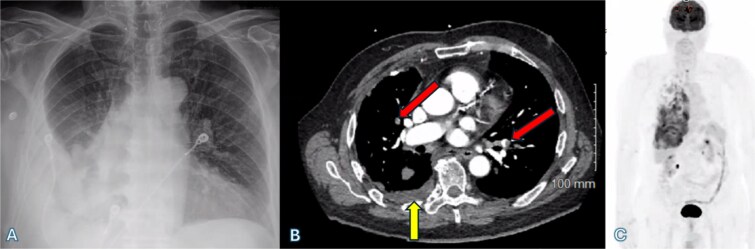
(A) Chest X-ray shows a right-sided pleural effusion. (B) Axial CT scan demonstrates pleural effusion (upward arrow) and bilateral pulmonary emboli (oblique arrows). (C) PET-CT scan shows right-sided pleural nodularity, metabolically active malignancy, and metastasis within the right pleural effusion.

An 80-year-old male with a history of chronic obstructive pulmonary disease (COPD), diabetes, thyroid cancer, and cutaneous melanoma presented to the emergency department with shortness of breath. The superficial spreading malignant melanoma on the left side of his back had been excised 12 years ago, with a Breslow thickness of 1.02 mm and a negative sentinel lymph node biopsy for metastasis. The patient remained under surveillance with no recurrence until 12 years later, when he presented with a pleural effusion in June 2024. Initially, he was treated for pneumonia and underwent pigtail catheter placement to drain a right-sided pleural effusion, with cytology and cultures showing negative results. He was discharged but returned a week later due to persistent shortness of breath and increased chest wall pain. A computed tomography (CT) scan revealed recurrent pleural effusion and right-sided pleural nodularity, raising concerns about malignancy. Given the short interval of effusion reaccumulation, he was transferred to our center for further evaluation and consideration for video-assisted thoracoscopic surgery. A right-sided pigtail catheter was placed again, and 20 ml of cloudy red fluid was drained, which was consistent with a culture-negative exudative effusion and nondiagnostic cytology. The CT scan revealed a small, right-sided loculated pleural effusion, numerous prominent and borderline enlarged mediastinal and bilateral hilar lymph nodes, nodularity extending along the fissures, solid nodularity within the posterior right upper lobe with the largest nodule measuring ⁓7 mm, and extensive bilateral pulmonary emboli (Fig. 1a and b), for which he was receiving heparin.

**Figure 2:**
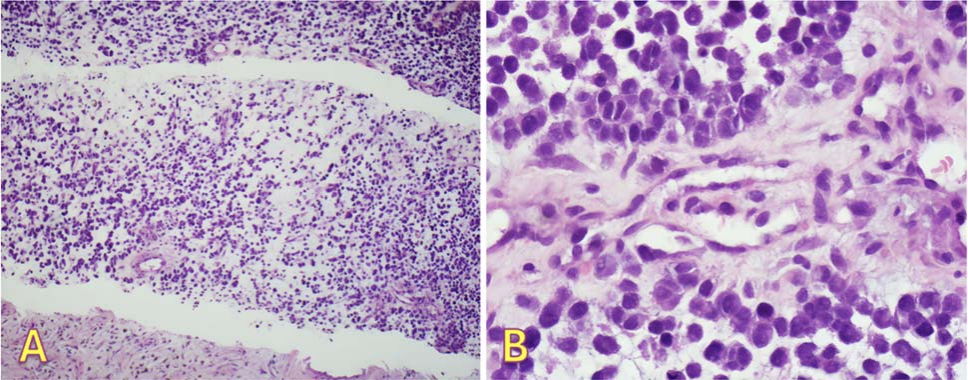
Right pleural biopsy: (A) sheets of metastatic cells H&E (×40), (B) round to oval malignant cells with eosinophilic cytoplasm, no melanin pigmentation was detected H&E (×400).

**Figure 3:**
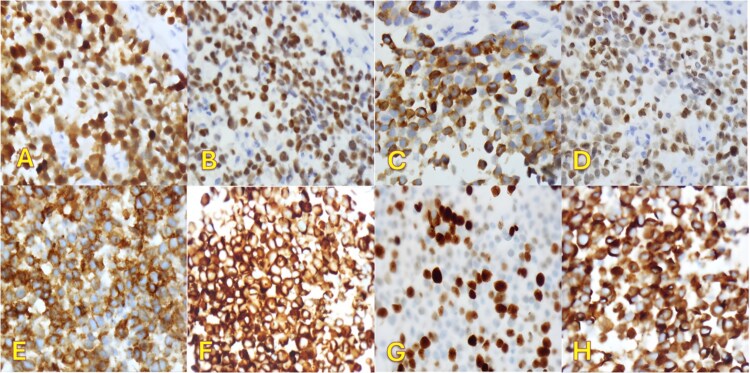
Immunohistochemical staining (×400). Pleural biopsy was strongly positive for (A) S100, (B) SOX10, (C) Melan-a, (D) MITF, (E) CD99, (F) BCL2, (G) Ki67, and (H) WT1.

An fluorodeoxyglucose positron emission tomography-computed tomography (PET-CT) scan showed highly suspicious metabolically active malignancy and associated metastasis involving the cluster of right upper lobe pulmonary nodules, right-sided pleural effusion/thickening, right-sided pleural nodularity extending along the fissure, multiple mediastinal lymph nodes and an avid lymph node within the post-thyroidectomy bed (Fig. 1c). As the patient was not a surgical candidate, the interventional radiologist performed an ultrasound-guided core biopsy, and pleurodesis with doxycycline was done through the pigtail catheter. The patient was discharged a week later with a stable X-ray and on Lovenox medication. His serum LDH level was elevated by 58 points, and Calcitonin was in the normal range. H&E staining revealed malignant cells with variable size, nuclear irregularity, and eosinophilic cytoplasm, indicating cancerous tissue within the pleura (Fig. 2). However, the exact primary origin of these malignant cells remained undetermined. Extensive immunostaining was performed to identify the source of the malignancy. Tumor cells were strongly positive for S100, SOX10, Melan-A, MITF, CD99, BCL2, WT, and Ki 67 at 40%–50%, confirming a diagnosis of metastatic melanoma (Fig. 3).

Upon molecular testing, NRAS Q61K (83.0% Variant Allele Frequency [VAF]) single nucleotide variant (SNV) mutation was identified, with no fusions or copy number variations detected. The first cycle of immunotherapy with Nivolumab/Relatlimab was initiated. Unfortunately, the patient’s condition progressively declined, and he passed away 2 weeks later.

## Discussion

This case is particularly interesting due to the rare presentation of pleural effusion and the metastasis occurring 12 years after the initial diagnosis. Most metastases typically occur within 3 years [4]. While late metastases are rare, they can arise even after decades [7, 8] and are associated with a poorer prognosis. In our patient, no recurrence was detected during the first 3–4 years, and the patient remained under surveillance until he presented with a malignant pleural effusion 12 years later. Chen *et al.* reported that among 130 cases of metastatic melanoma in the chest, only three cases (2%) resulted in malignant pleural effusion [4]. D’Ambrosio *et al.* described a case of pleural melanoma metastasis that occurred 4 years after the initial diagnosis [9]. Yu *et al.* reported a case of melanoma pleural effusion after a 6-year interval [5]. Sotelo *et al.* noted pleural metastasis 14 years after the initial cutaneous presentation, and Morales *et al.* presented an interesting case of metastatic melanoma to the pleura diagnosed 21 years after the original resection [10].

Metastases to any location, including the pleura, with an elevated LDH level have the worst prognosis, with a 1-year survival rate of 33%. Atypical cells, especially those without melanin pigments, can mimic malignant mesothelioma or lung adenocarcinoma, so the key to diagnosis is to consider metastatic melanoma in the differential diagnosis [11].

A new or recurrent pleural effusion has a broad differential diagnosis, and the evaluation includes the inspection of the gross appearance of the fluid, differentiating exudative from transudative, and cytology analysis. A few studies described the rare black color exudate due to the presence of melanocytes in the fluid [12]**.** The pleural effusion is generally small, unilateral or bilateral, and can be associated with pulmonary nodules and adenopathy [4]. In our case, the effusion was small and cloudy. The cloudy appearance of the effusion suggested an exudative process, but melanoma was not initially suspected due to the complete absence of melanin pigments. However, in cases of undifferentiated neoplasms without clear lineage, melanoma should be considered despite its rarity. Bilateral Pulmonary embolism (PE) was another finding in our patient. PE in patients with malignant melanoma (MM) often occurs without clinical symptoms and is indicative of advanced disease and a poorer prognosis. Several studies have shown that hypercoagulability measured by serum D-Dimer levels can predict tumor aggressiveness and survival in MM patients [13].

The LDH level in our patient was higher than normal. High serum and pleural fluid LDH levels are also less favorable prognostic indicators [6]. Since most melanoma recurrences or metastases occur within the first few years, the general guidelines suggest limiting imaging after 3–5 years [14]. The absence of regular imaging doesn’t mean a lack of monitoring. Long-term monitoring and extended imaging for high-risk patients are vital for catching late recurrence, as seen in our case. Melanomas with NRAS Q61k mutation are less common and more aggressive, with a higher risk of recurrence and metastasis [15]. However, a high VAF of 83% might make the tumor more recognizable to the immune system and potentially more responsive to immunotherapies like immune checkpoint inhibitors (ICIs) [16]. This case, featuring a rare pleural metastasis from cutaneous melanoma occurring more than a decade after initial diagnosis, highlights the importance of maintaining a high suspicion for metastatic melanoma to prevent delays in diagnosis and improve treatment outcomes.
